# Variations in small-scale movements of, *Rousettus aegyptiacus,* a Marburg virus reservoir across a seasonal gradient

**DOI:** 10.1186/s12983-023-00502-2

**Published:** 2023-07-18

**Authors:** Matthew R. Wood, J. Low de Vries, Jonathan H. Epstein, Wanda Markotter

**Affiliations:** 1 Present Address: Centre for Viral Zoonoses, Department of Medical Virology, University of Pretoria, Pretoria, South Africa; 2 EcoHealth Alliance, New York, NY USA

**Keywords:** Chiroptera, Contact risk, Egyptian rousette bat, Movement patterns, Zoonoses

## Abstract

**Background:**

Bats are increasingly being recognized as important hosts for viruses, some of which are zoonotic and carry the potential for spillover within human and livestock populations. Biosurveillance studies focused on assessing the risk of pathogen transmission, however, have largely focused on the virological component and have not always considered the ecological implications of different species as viral hosts. The movements of known viral hosts are an important component for disease risk assessments as they can potentially identify regions of higher risk of contact and spillover. As such, this study aimed to synthesize data from both virological and ecological fields to provide a more holistic assessment of the risk of pathogen transmission from bats to people.

**Results:**

Using radiotelemetry, we tracked the small-scale movements of *Rousettus aegyptiacus,* a species of bat known to host Marburg virus and other viruses with zoonotic potential, in a rural settlement in Limpopo Province, South Africa. The tracked bats exhibited seasonal variations in their movement patterns including variable usage of residential areas which could translate to contact between bats and humans and may facilitate spillover. We identified a trend for increased usage of residential areas during the winter months with July specifically experiencing the highest levels of bat activity within residential areas. July has previously been identified as a key period for increased spillover risk for viruses associated with *R. aegyptiacus* from this colony and paired with the increased activity levels, illustrates the risk for spillover to human populations.

**Conclusion:**

This study emphasizes the importance of incorporating ecological data such as movement patterns with virological data to provide a better understanding of the risk of pathogen spillover and transmission.

**Supplementary Information:**

The online version contains supplementary material available at 10.1186/s12983-023-00502-2.

## Background

Movements of animals are linked to changes in their internal state or environmental conditions that induce a critical response to satisfy essential requirements for survival [1]. Aside from the myriad of factors that may affect movement patterns such as seasonal changes, predation pressure or the need to find a mate [2], the availability of limiting resources such as food, water and suitable habitats is generally considered the most prominent determinant of movement patterns [3, 4]. Bats, belonging to the order Chiroptera, are very diverse with 1456 reported species [5] and are unique as the only mammals capable of self-powered flight [6]. Flight enables bats to traverse larger areas compared to similar-sized terrestrial species and cross ecological or geographic barriers that would typically inhibit movements [7, 8]. The Egyptian rousette bat (ERB), *Rousettus aegyptiacus*, is a medium-sized fruit bat (mean body mass—120 g; mean forearm length: 92 mm) [9] with a wide geographic distribution across Africa and into the Middle East [10]. They roost in large colonies that can range from a few hundred to several thousand individuals [9] and are typically frugivorous, although there is evidence of them feeding on pollen, flowers, leaves and insects [11, 12]. Egyptian rousettes are capable of long-distance flights [13] and possess internal navigation capacities that enable them to locate distant resources [14]. They have been recorded altering their movement patterns according to fruit availability across a seasonal gradient [15–17]. In Egypt, *R. aegyptiacus* decreased their home range size during seasons with food scarcity and likely concentrated their activities around a few reliable resource patches [17]. A similar trend was observed in Cyprus where ERBs had smaller home ranges in summer when resource availability and the diversity of fruiting trees was low [16]. During winter, however, a higher diversity of trees was fruiting and overall fruit availability increased, therefore, the bats could target a wider variety of food sources and expand their ranges [16]. In South Africa, Barclay and Jacobs [15] demonstrate that, although ERBs foraged in human-developed areas, they preferentially targeted natural food sources during seasons with high resource availability.

Chiroptera is the second-most speciose order after Rodentia and was theorised to carry a higher proportion of zoonotic viruses per species than any other mammalian order [18]. Although, Olival et al. [19] and Mollentze and Streicker [20] suggested that the number of zoonotic viruses was proportional to what would be expected given their species diversity. It has been hypothesised that the cause for their prominence as viral hosts is linked to key ecological traits that increase their suitability as viral reservoirs [18, 21, 22]. These traits include highly gregarious aggregations in some species that increase the chance for contact between species or with conspecifics, and long lifespans [18, 21, 22]. Furthermore, flight is an important factor as it enables widespread movements, and the broad geographic ranges for some species may facilitate contact and potential viral sharing with species across a wide range [18] and, in some cases, across national borders [7, 23]. Bats are associated with several viral families, including *Filoviridae*, *Paramyxoviridae* and *Coronaviridae* as well as lyssaviruses from the *Rhabdoviridae* family, that have already demonstrated spillover and the potential for high pathogenicity [24, 25]. Within these families, there are notable examples where bats have been identified as the virus reservoir hosts namely Marburg virus [26], Nipah virus [27] and Hendra virus [28, 29]. The Egyptian rousette has been identified as the reservoir host for Marburg virus, which, like Ebola virus, causes a fatal haemorrhagic fever in people [26, 30, 31]. ERBs have also been associated with paramyxoviruses, lyssaviruses and coronaviruses, several with zoonotic potential [32–34]. Their role as a reservoir host for viral zoonoses such as Marburg virus and association with human outbreaks in East and West Africa, as well as their association with other viruses with zoonotic potential, highlights the need to better understand the potential for ERBs to transmit these viruses to humans living in close association with colonies [15, 17]. A colony of ERBs from Matlapitsi cave, South Africa has been associated with Marburg virus [30] and several paramyxoviruses with zoonotic potential [33, 35]. Seasonal patterns of viral shedding have been identified, with peaks occurring in the dry season, and July specifically [30, 33, 35]. The hypothesised reason for this is waning maternal antibodies in juveniles which leaves them susceptible to infection and therefore, viral shedding [32]. The hypothesised routes of viral shedding for Marburg virus are through urinary excretions and saliva [36, 37], although nearly all cases of spillover of Marburg virus from *R. aegyptiacus* to humans occurred when people entered cave roosts directly [37]. Outside of roost sites, the risk of direct human contact with ERBs is limited but indirect transmission through materials contaminated with saliva or urine is possible [36]. ERBs are known to discard partially chewed fruit on the ground around fruiting trees [38] and this poses a theoretical route of exposure to viruses if other animals or people pick up these fruit spit-outs, especially for Marburg virus [39]. Viable Marburg virus has been detected on experimentally inoculated fruit 6 h after inoculation, highlighting discarded fruit as a plausible route for transmission [37].

A relatively novel attitude in the field of zoonoses and virus transmission has been to integrate expertise from multiple fields including virology, ecology, anthropology and sociology to provide a better understanding for risk assessments [40, 41]. Virological studies, while highlighting important components such as viral prevalence, diversity and excretion dynamics, lack the ecological perspective that could provide context and potential scale for disease transmission risk [23, 40]. Similarly, while focused ecological studies can provide insight into a species’ interactions with other species and potential spillover hosts, without the virological background, those data are effectively meaningless in the context of disease risk assessments [40]. The integration of the two fields, however, can provide a more holistic understanding for risk assessments. For instance, movement data can help identify key areas in the landscape that are favoured by a study species and may provide evidence of spatial overlap, and by extension, the likelihood of contact between humans, wildlife and livestock species [23, 40, 42]. Furthermore, movement studies may identify patterns of interconnectedness between separate populations which can influence pathogen dynamics, maintenance and evolution within a system [43]. If the movement data are obtained for species that are known hosts of potential zoonotic pathogens and paired with data regarding pathogen prevalence, excretion and shedding dynamics, researchers can extrapolate the data to assess the risks of spillover events or disease transmission [40, 44]. Spillover events are rare and certain processes must occur before spillover can take place [44, 45], but the alteration of land use that disrupts natural processes has been shown to be a crucial step [46, 47]. These disruptions can alter pathogen transmission dynamics and potentially open up new niches for transmission [48–50]. Furthermore, outbreaks have been shown to be more likely to occur in areas with high species diversity and interactions at human, livestock and/or wildlife interfaces [51–53]. Such interfaces are occurring with increasing regularity as anthropogenic expansion encroaches on natural areas, and this may influence the degree of contact between a reservoir species and potential susceptible hosts, further increasing the risk of spillover [54, 55].

This study aimed to assess the movement patterns of ERBs roosting near a rural human population in South Africa. Specifically, we focused on: (1) determining whether their nightly foraging movements varied on a seasonal basis linked to different fruiting periods for natural or cultivated fruit trees and, (2) whether their movement patterns overlapped with human presence, creating opportunities for contact and potential spillover of zoonotic viruses. We predict that the movement patterns of *R. aegyptiacus* will exhibit seasonal patterns in line with fruit tree phenology and their foraging areas will include residential areas which may increase the risk of contact and pathogen spillover.

## Results

Tracking was performed for 95 nights totalling 627 tracking hours across the 12-month study period. We obtained 930 locations for the 26 tagged bats (Table 1) and additionally recorded 295 unique locations for bat sightings throughout the study period (Additional file 1).

**Table 1:** Study animals

Bat ID	Sex	Age	Reproductive status	Body mass (g)	Forearm length (mm)	Number of locations	Number of months tracked
UP21-02	F	SA	NP	75	84.3	55	6
UP21-03	M	A	S	127	92.2	39	4
UP21-04	M	A	S	130	93.8	21	3
UP21-05	M	SA	NS	82	83.5	34	5
UP21-06^a^	F	SA	NP	82	85.3	25	3
UP21-07	F	A	NP	104	89.7	38	5
UP21-08	F	A	NP	112	94.2	30	3
UP21-09	F	SA	NP	88	84.6	30	3
UP21-10	F	A	NP	133	90.0	34	4
UP21-11	M	A	S	119	93.2	30	3
UP21-12	M	A	S	113	92.8	28	4
UP21-13^a^	M	SA	NS	88	84.7	22	2
UP21-14	M	A	NS	127	90.4	13	2
UP21-15	F	A	NP	74	90.2	13	2
UP21-200	M	A	S	128	94.6	52	6
UP21-201	F	A	NP	122	94.0	55	6
UP21-202	M	A	NS	113	93.8	55	6
UP21-203	M	A	NS	107	89.9	39	4
UP21-204	F	A	NP	96	90.3	57	6
UP21-205	M	SA	NS	95	88.8	46	5
UP21-206	F	A	NP	109	90.3	20	4
UP21-207	M	A	NS	100	89.2	31	4
UP21-208	M	A	S	138	95.8	37	5
UP21-209	F	A	NP	97	92.4	65	6
UP21-210	M	A	S	137	89.5	35	5
UP21-211	M	A	S	126	93.1	27	4

Summary of tagged Egyptian rousette bats, sex, age, reproductive status, body mass, forearm length, the number of tracked locations and number of months tracked

*F* female, *M* male, *A* adult, *SA* subadult, *NP* non-pregnant, *S* scrotal, *NS* non-scrotal

^a^Discarded tags recovered and redeployed on UP21-14 and UP21-15

Model selection showed that the best-performing model included habitat type, activity and the interaction between habitat type and activity as fixed effects with month as the random effect. Although the model that included season was within ΔAICc < 2 (corrected Akaike’s Information Criterion) and was, therefore, treated as a competing model (Additional file 2).

Habitat type and activity independently had a significant influence on the number of locations obtained each month as well as the interaction between habitat type and activity (Table 2). For the competing model where season was included, the results remained the same and season was shown not to have a significant effect on the number of locations obtained (z = 0.952; *p* = 0.34).

**Table 2:** Model output

Coefficient	Estimate		Std.error		C.I		z-score		*p* value	
Intercept	2.61		0.12		2.49 – 2.73		22.46		< 0.001	
Habitat type										
Agricultural	Ref lev*									
Natural	0.28		0.10		0.18–0.38		2.72		< 0.05	
Residential	− 0.71		0.13		− 0.84 to − 0.58		− 5.30		< 0.001	
Activity										
Commuting (F)	Ref lev									
Foraging (NF)	− 0.42		0.12		− 0.54 to − 0.30		− 3.44		< 0.001	
Habitat.Natural*Activity.NF	0.50		0.15		0.35–0.65		3.27		< 0.05	
Habitat.Residential*Activity.NF	0.53		0.19		0.44–0.72		2.75		< 0.001	

Summary of best-performing model output including estimates, standard error, 95% confidence intervals, z-scores and *p* values

*Reference level variable

Throughout the study period, the mean number of locations obtained from natural areas was significantly higher than from agricultural and residential areas, whereas the number of locations obtained from agricultural areas was significantly higher than from residential areas. Overall, bats were recorded commuting more often than foraging. However, when the different landcover types were considered as well the highest number of locations was obtained for foraging bats in natural areas followed by commuting bats in natural and agricultural areas. The lowest number of locations were recorded for commuting bats in agricultural areas although this was not significantly different compared to locations recorded in residential areas for either activity (Table 3). Bats were mostly foraging within diverse fruiting trees present in natural areas but did make use of the spaces between treelines as flyways for commuting. Within agricultural areas, there were very few foraging locations and therefore, bats were predominantly using these areas for commuting. Residential areas offered alternative fruit sources and foraging sites for the bats during the dry season. There are no known alternative roost sites within the valley and we recorded no evidence of bats utilising temporary shelters or roosts. The separate linear model assessing tracking effort showed that the number of active tags per month (β = 5.15, *p* < 0.05) had a significant, positive influence on the number of locations recorded. The number of nights (β = 10.34, *p* = 0.14) and hours tracked each month (β = − 0.05, *p* = 0.95) had no influence. Therefore, it is reasonable to assume that our results were not biased by tracking effort.

**Table 3:** Habitat type and activity interaction

Comparison	Estimate	SE	*p* value
Agriculture.F—Natural.F	− 0.2759	0.1015	0.0716
**Agriculture.F > Residential.F**	0.7050	0.1330	**0.001**
**Agriculture.F > Agriculture.NF**	0.4173	0.1214	**0.0077**
**Agriculture.F < Natural.NF**	− 0.3573	0.0997	**0.0046**
**Agriculture.F > Residential.NF**	0.5925	0.1282	**0.001**
**Natural.F > Residential.F**	0.9808	0.1276	**0.001**
**Natural.F > Agriculture.NF**	0.6931	0.1154	**0.001**
Natural.F – Natural.NF	− 0.0814	0.0924	0.9511
**Natural.F > Residential.NF**	0.8684	0.1226	**0.001**
Residential.F—Agriculture.NF	− 0.2877	0.1440	0.3434
**Residential.F < Natural.NF**	− 1.0622	0.1263	**0.001**
Residential.F—Residential.NF	− 0.1125	0.1498	0.9754
**Agriculture.NF < Natural.NF**	− 0.7746	0.1139	**0.001**
Agriculture.NF—Residential.NF	0.1752	0.1395	0.8091
**Natural.NF > Residential.NF**	0.9498	0.1212	**0.001**

Pairwise comparisons of the interaction between habitat type and activity for the number of monthly locations, including the estimates, standard errors and *p* values

Significant comparisons given in bold

**Fig. 1:**
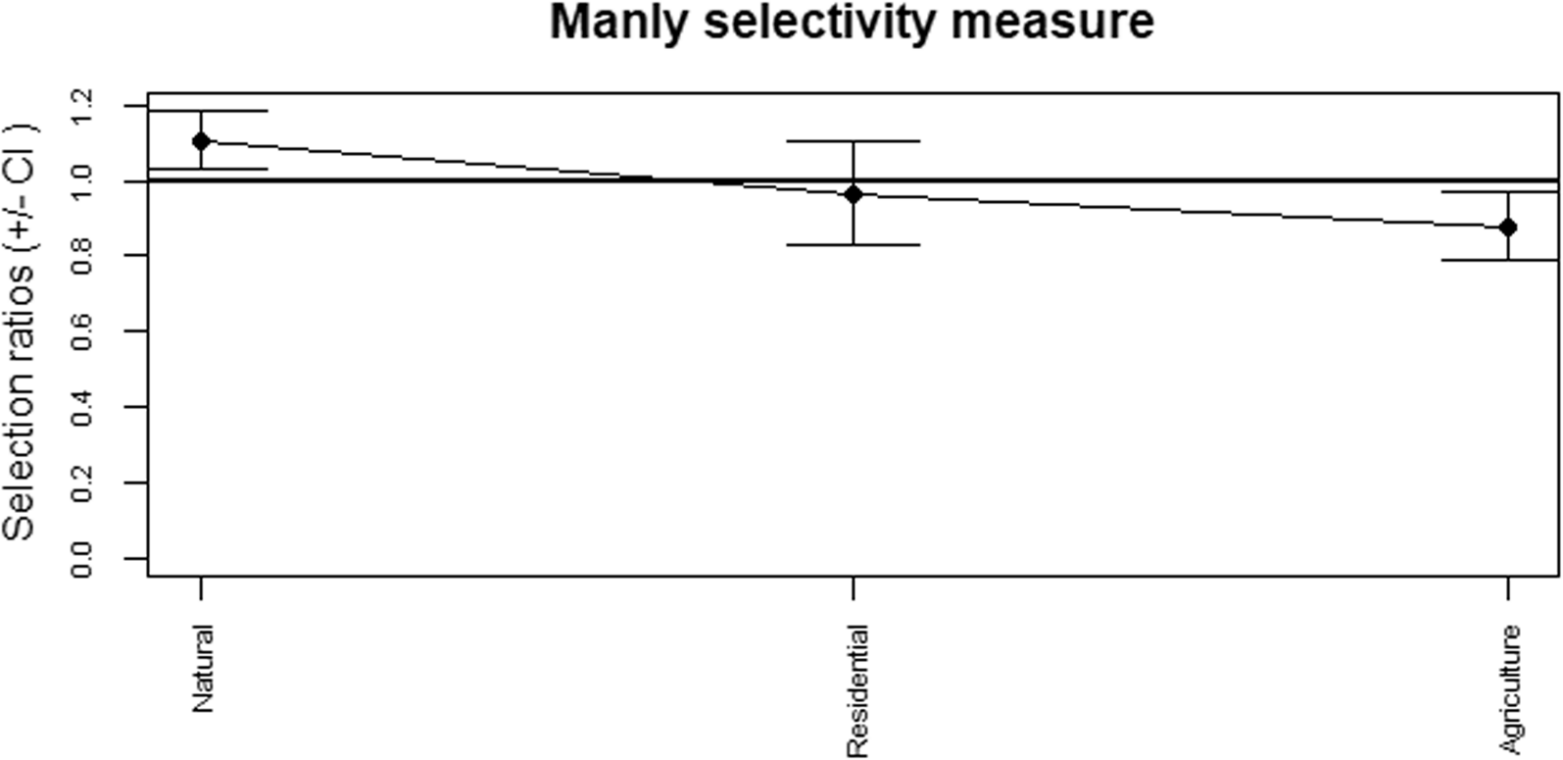
Habitat selection during the study period. Manly selectivity measure with 95% confidence intervals for habitat selection throughout the study period. Where standard error bats overlap with the solid line, it indicates no selection. Selection ratios > 1 indicate positive selection; < 1 indicates negative selection

The above analyses, however, only utilized the mean number of locations and did not consider the effect of habitat availability within the study area. Habitat availability was defined as the proportional area size for the different habitat types identified from the South African National Landcover (SANLC) database out of the total area for the study site. When the proportion of habitat availability was accounted for, there was a non-random selection of different habitats. The terms ‘positive selection’ and ‘negative selection’ were used when referring to habitat usage rather than ‘preference/avoidance’ or ‘presence/absence’ as these latter terms can be ambiguous and difficult to accurately quantify [56]. For instance, determining the avoidance of an area can, similarly to absence records, be incorrectly recorded if the animal is not detected despite its presence in the area, a feature we may likely have encountered, given the difficulty of tracking small, flying animals such as bats [57]. Natural areas were consistently and significantly selected for more than their relative availability. By contrast, the number of locations obtained in agricultural areas was significantly lower than expected given its proportional availability within the study area (Table 4). There was no pattern for positive or negative selection in residential areas. The selectivity plot for habitat selection throughout the year illustrates the positive and negative selection for natural and agricultural areas respectively (Fig. 1).

**Table 4:** Overall habitat selection

	Used	Available	w.i.^a^	SE w.i	*p* value
Residential	0.191	0.198	0.966	0.065	0.597
Agricultural	0.303	0.345	0.879	0.044	< 0.0167
Natural	0.505	0.457	1.107	0.036	< 0.0167

Summary table of habitat selection ratios for the full study period with proportional usage, proportional availability, selection ratios (w.i.) for habitat type and the corresponding *p* values. Significant *p* values were determined after Bonferroni adjustment (significant *p* value < 0.0167)

^a^w.i. > 1 indicates positive selection; w.i. < 1 indicates negative selection

**Fig. 2:**
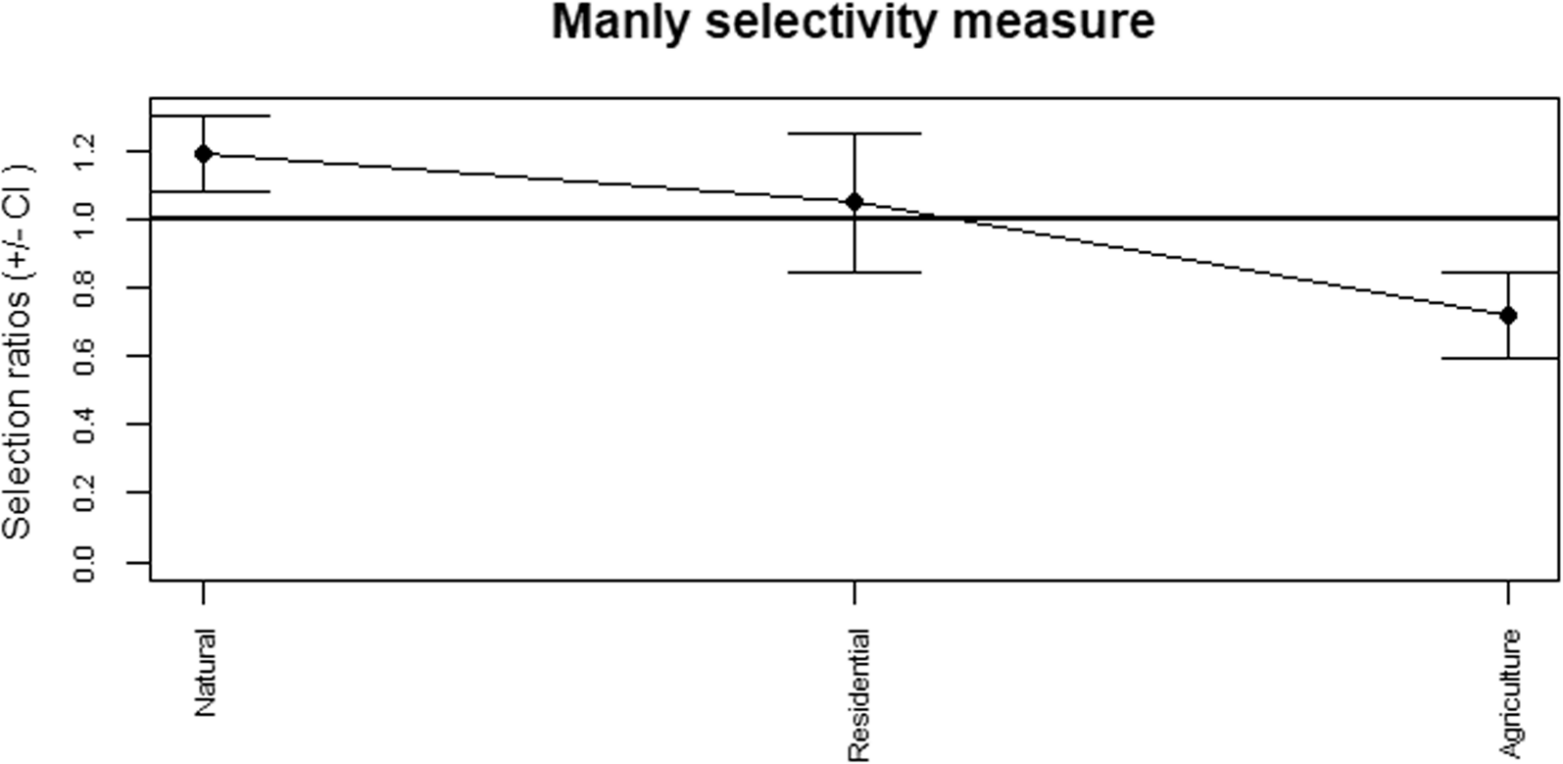
Habitat selection for non-flying activities during the study period. Manly selectivity measure with 95% confidence intervals for non-flying activities throughout the study period. Where standard error bats overlap with the solid line, it indicates no selection. Selection ratios > 1 indicate positive selection; < 1 indicates negative selection

When habitat selection was assessed for different activities, there were no clear trends for significant selection of any habitats for commuting. However, there was a clear positive selection for natural areas and negative selection of agricultural areas for foraging activities (Table 5). The selectivity plot follows a similar trend to the one for the full study period with all locations, although the negative selection for agricultural areas was even stronger for foraging activities (Fig. 2). There was no evidence of habitat selection for males or females.

**Table 5:** Habitat selection for foraging activities

	Used	Available	w.i	SE w.i	*p* value
Residential	0.208	0.198	1.049	0.096	0.61
Agricultural	0.248	0.345	0.718	0.059	< 0.0167
Natural	0.544	0.457	1.192	0.051	< 0.0167

Habitat selection ratios for foraging activities with the proportional usage, proportional availability, selection ratios (w.i.) for habitat type and the corresponding p-values. Significant *p* values were determined after Bonferroni adjustment (significant *p* value < 0.0167)

The patterns of usage of residential areas varied across the study period with the overall highest activity occurring in July 2021 and the lowest in January 2022 with the same pattern for foraging and all locations (Additional file 3). Important to note was the trend for a decrease in the use of residential areas after October, despite an increase in fruit availability estimates for the mango and banana trees we monitored in residential areas (Additional file 4), both of which are known food sources for ERBs [38]. Bats likely did not need to rely on mangoes or bananas during this period given the patterns of increased fruit availability for our proxy trees within natural areas as well and observations of bats feeding in *Ficus sycomorus, F. sur* and *Ekebergia capensis*.

**Fig. 3:**
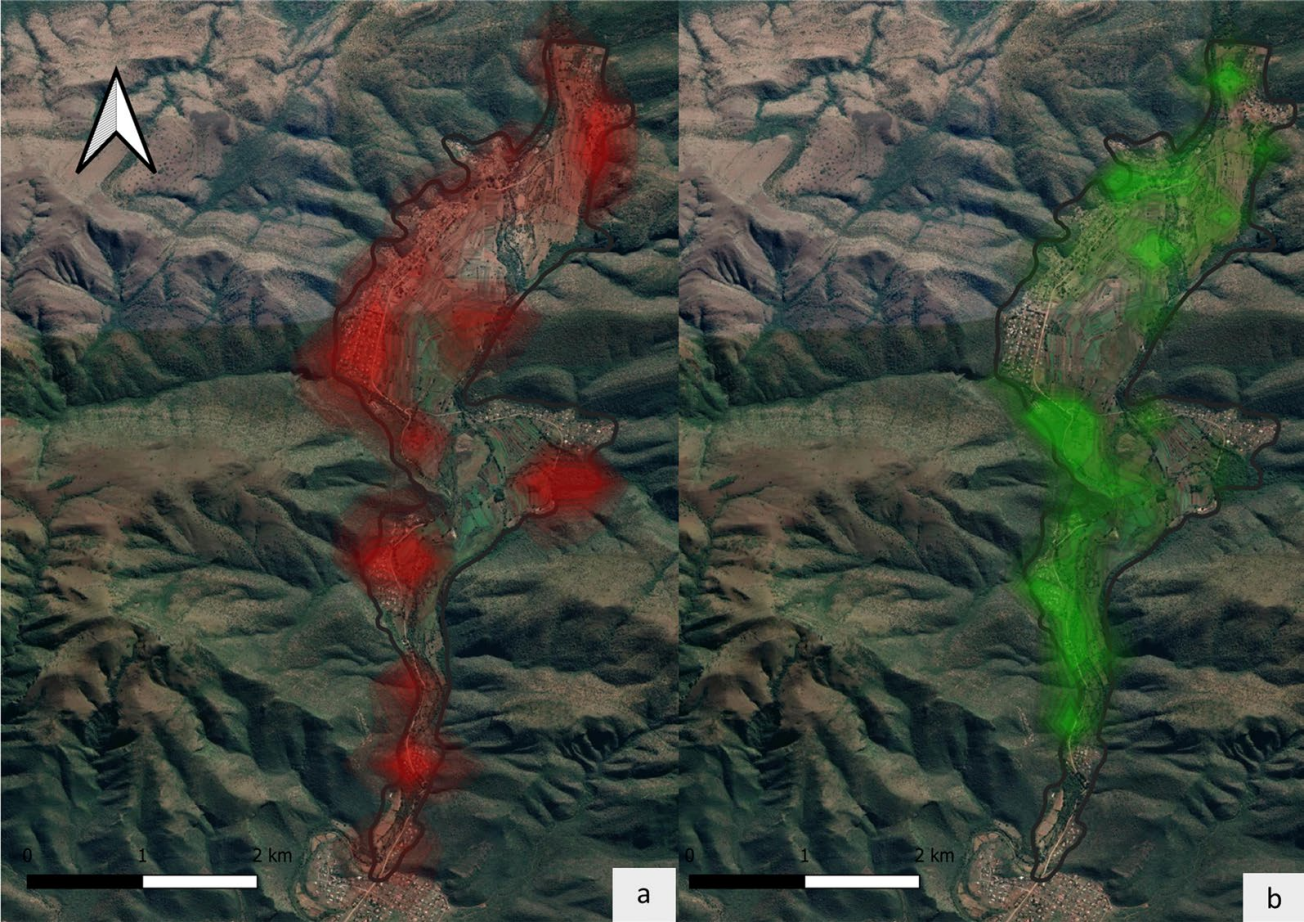
Utilization distributions for non-flying activities. Kernel density estimates showing utilization distributions for non-flying locations during July 2021 (**a**) and January 2022 (**b**). Darker areas depict regions with more intense levels of activity

The utilization distributions between seasons further supported this trend as usage of residential areas was significantly greater for foraging activities during the dry than the wet season (*t*_(5)_ = − 2.70, *p* < 0.05). To expand on these trends, we further compared the utilization distributions for foraging activities between the months with the highest (July 2021—Fig. 3a) and lowest activity levels within residential areas (January 2022—Fig. 3b) showing that the proportional usage of residential areas was significantly greater in July than January.

Not only that, but the proportional area size within residential areas for July was significantly larger than the other habitat types for both January and July (Additional file 5). Furthermore, the proportional usage of residential areas during January was significantly less than that observed for the other habitat types in January and July, although its usage was on par with natural areas in July (Additional file 6). This suggests that bats preferentially utilise residential areas for foraging during the dry season when natural fruit sources are scarce, but resort back to natural areas once they start fruiting again in the wet season. This pattern is of significance for July specifically as it is one of the months previously identified as a high-risk period for potential pathogen shedding as it coincides with the waning of maternal antibodies within juveniles in the colony [30] further emphasising the risk of pathogen transmission during this period.

## Discussion

There have only been two previous movement studies for ERBs in South Africa [13, 15] and this was the first study to assess the movements of ERBs in the context of potential spillover in a rural community. This species, and this colony specifically, have been identified as hosts for viruses with zoonotic potential [33, 35] including Marburg virus [30, 39] which is responsible for several outbreaks with high fatality rates in Eastern and West Africa [58, 59]. This study identified trends in seasonal movement patterns including periods of high contact risk between bats and people that could constitute a risk of pathogen spillover and transmission. In a landscape characterised by large expanses of agricultural areas and small residential settlements, we show evidence of habitat preferences for foraging and commuting. Furthermore, there was a trend for increased activity within residential areas during the dry, winter months, specifically July, which has been identified as a critical period for viral shedding for paramyxoviruses [33, 35].

Bats utilised all habitat types available to them but showed a preference for natural areas and relative disuse of agricultural areas. Natural areas were used for commuting and foraging with equal likelihood; however, agricultural areas were used for commuting significantly more often than foraging. They have been observed utilising open areas as flyways when traversing large distances [14], but at smaller scales have been reported avoiding large open fields for foraging [16]. Similarly, lesser short-nosed fruit bats, *Cynopterus brachyotis*, and frugivorous phyllostomids only used plantations as flyways when few to no suitable foraging sites were present [60, 61]. The agricultural fields in our study site are predominantly used for vegetable crop production or livestock grazing with few sparsely distributed fruiting trees and therefore, provide little incentive for fruit bats as foraging sites.

Residential areas were used as frequently as would be expected by random selection with no preference for foraging or commuting activities. However, bats used significantly larger proportions of residential areas than other habitat types for foraging during the dry season. The trees we monitored for fruit availability within natural areas had little to no fruit during the dry season, whereas the trees within residential areas, especially bananas, maintained moderate levels of fruit availability. Therefore, bats likely relied on residential areas for foraging during the dry season when food was scarcer in natural areas. Egyptian rousette bats have been shown to opportunistically exploit human-modified areas as foraging sites [16, 42] and were observed altering movement patterns specifically to target orchards or cultivated crops when overall resource availability is low [17, 62]. Unfortunately, the spatial resolution of the movement data was not high enough to enable pinpointing exact locations utilised by the bats. As such we cannot quantify whether specific areas or locations within residential areas are targeted more frequently, although it is unlikely that the activities of bats and humans overlapped on a temporal scale as farming and harvesting activities are ceased before sunset.

Contact between fruit bats and potential spillover hosts is likely more prevalent for species that are active at night since their periods of activity overlap. However, indirect contact with diurnal species may also occur through faecal or urinary excretions or fruit discarded by bats while foraging [36]. Egyptian rousette bats typically chew fruits to extract the juices from the pulp and then discard the remainder of the fruit [62]. These ‘spit-outs’ may be contaminated with saliva and recent research on captive ERBs showed that viable Marburg virus was still detected on artificially inoculated fruits 6 h post-inoculation [37]. This suggests that early morning activities of people or livestock could feasibly overlap with periods where the virus is still viable. There has been no evidence of people or animals picking up or eating the fruit scraps discarded by *R. aegyptiacus*, but there are community meetings held underneath one *F. sycomorus* that is a frequent foraging site. There is also a dip-tank for cattle underneath the same tree. Both of these constitute supplementary sites where humans, livestock and bats are regularly experiencing spatial overlap and therefore, contact and spillover risk. Although, there has been no evidence of direct contact, previous spillover events and outbreaks for other bat-borne zoonoses have occurred through indirect contact. For example, Hendra virus spillover was predicted to have occurred through urinary, faecal or salivary contamination of drinking water or grazing areas in horse pastures [63]. Similarly, Nipah virus outbreaks likely stemmed from people drinking palm sap that had been contaminated by bat urine and saliva [64].

Previous outbreaks of bat-borne zoonoses were associated with the overlap in distributions of bats, people and livestock species that served as intermediate hosts [48, 63–65]. Therefore, our evidence of bats utilising residential areas for commuting and foraging demonstrates the risk of contact, exposure and potential future spillover. Specifically, July represents the period of greatest risk, not only because activity levels in residential areas are highest, but also because July has been identified as a critical period for increased potential shedding of Marburg virus [30] and paramyxoviruses [35]. The reason July is considered a critical period for viral shedding is that it coincides with waning maternal antibodies in juvenile *R. aegyptiacus* [32]. This translates to a higher proportion of susceptible individuals in the colony, higher levels of viral transmission and consequently an increased risk of viral shedding [32].

And yet, despite the historic proximity between the colony and human populations, evidence of spatial overlap between bats and humans and times during the year when the risk of viral shedding is increased, there have never been any recorded spillover events in the area. One possibility is that clinical cases simply were not documented as the nearest hospital is approximately an hour’s drive away and transport is not easy to come by so patients may have opted for local care. The other possibility is that, to date, there have been no spillover events which leads to a follow-up question: why have no spillover events occurred? Plowright et al. [44] outlined a series of key factors linked to viral and host ecology that may act as barriers to spillover and only if all of these barriers are overcome by the pathogen, can spillover occur.

A preliminary consideration is whether the distribution of the reservoir host overlaps with the potential spillover host. From our tracking data, we know that the distribution of fruit bats from Matlapitsi cave overlaps with humans within our study area, specifically during July when the risk of viral shedding is increased [30, 35]. However, we also observed an overall decrease in bat presence within the valley during winter with bats typically detected just after sunset and then only again just before sunrise. The colony size for *R. aegyptiacus* in Matlapitsi is known to vary throughout the year with the lowest numbers recorded during winter [62], however, it is unknown exactly where the bats go during winter. One potential site is a sinkhole in the Lekgalameetse Nature Reserve which supports a colony of *R. aegyptiacus*, although none of our tracked bats were detected there. For the bats that remain in Matlapitsi cave during winter, some of them may be foraging outside of the valley and could feasibly be travelling anywhere within a 24 km radius of their roost site to forage [13]. Therefore, any residential areas, fruit orchards or natural regions within this range could be targeted for foraging and therefore, the risk of contact and overlap with humans is potentially not limited to Fertilis. Distant foraging sites are plausible as the Greater Tzaneen area, just north of our study site, is a prominent producer of a variety of fruit products in South Africa with many orchards as supported by the research of Tshilowa [66] and there are several small towns nearby where subsistence fruit production also occurs.

Subsequent knowledge of the pathogen prevalence in the reservoir host can also help gauge the risk of contact between the reservoir host and susceptible recipient hosts within its distribution. These data are not available for our focal colony; however, previous longitudinal studies have identified periods throughout the year with increased levels of viral RNA (ribonucleic acid) detection [33, 35] as well as decreased viral antibody seropositivity [30, 39] both of which may correspond to periods of increased likelihood for viral shedding. Routes of potential viral excretion and pathogen survival rates outside of the reservoir can identify possible routes of contact and risk of exposure. Routes of viral shedding have been identified through urine, faecal excreta, faecal material from rectal swabs and saliva from oral swabs for Marburg virus and a variety of paramyxoviruses [33, 35, 39, 67]. Therefore, potential routes of spillover and transmission could be through contact with materials contaminated by urine or saliva [37].

Finally, the internal characteristics of potential spillover hosts influence the probability and severity of infection. Data for these aspects of the spillover process are unknown for the population in our study area, although previous, large-scale Marburg virus outbreaks have occurred in other parts of Africa [36, 58, 68] emphasising the potential for a spillover event. The other viruses associated with this Egyptian rousette population are not known to cause infection in humans but are closely related to viruses with zoonotic potential [33, 35] and therefore, are also important to consider when assessing the overall risk of potential viral spillover.

If the above factors are considered, according to Plowright et al. [44], the risk and potential for spillover are high yet there are some other possible explanations for the absence of a spillover event. It was suggested that the lack of local disease, despite the presence of zoonoses in the bat colony, could be attributed to the lack of activities such as hunting bats for bush meat or entering the cave for guano mining [35]. All previous human infections of Marburg virus can be traced back to cave entries, sharing living areas with reservoir hosts or direct exposure to infected specimens [37, 68]. While people do still enter the cave, it is not a common practice and therefore the most likely interface for contact with bats would be outside the cave where external factors or asynchronous windows of activity may prevent exposure and spillover. The possibility for viable Marburg virus to persist on discarded fruit [37] notwithstanding, the limited or complete lack of temporal overlap between bat and human activities is likely an important factor confounding the spillover process [42]. It may also be the case that the viruses with zoonotic potential have not yet evolved the specific mechanisms required for cell entry, replication and human infection [55]. Alternatively, the spillover process may be incomplete and that transmission to an intermediate host may have occurred rather than direct transmission to humans. We observed close interactions between bats and other wildlife species while foraging, especially bushbabies and genets, that could plausibly facilitate exposure and spillover. Genets have been observed directly preying on *R. aegyptiacus* in the Fertilis valley, while bushbabies are regularly observed foraging in the same fruit trees as the bats. These were, however, based on incidental observations and as such the frequency with which these interactions occur is unknown and no viral surveillance of wildlife species has yet been performed in the area. Therefore, it should form part of future investigations to determine whether transmission to an intermediate host has occurred.

As this study was solely focused on assessing the spatial overlap and risk of contact between bats and people within Fertilis, we did not attempt to track the bats’ movements outside of the study area. These movement trends are unknown but Global Positioning System (GPS) tags could be used to determine the movements of *R. aegyptiacus* outside of the Fertilis valley. These movement data could be especially valuable during the dry season to identify key foraging sites and possible alternative roost sites outside of the valley. A further drawback of the study was the lack of virological data for the specific bats that were tracked. There is evidence that *R. aeyptiacus* from Matlapitsi cave are hosts of Marburg and potentially other viruses with zoonotic potential [30, 33, 35] and the risk of contact has already been established through the findings of this study. Virological data for individual tracked bats may have provided insight into the real-time risks of viral spillover, although we did not expect the infection status of bats to affect their behaviours and movement patterns [69].

## Conclusion

This study provides insight into the seasonal movement patterns of a known viral host, an aspect which, up until recently, was often not an integral part of viral biosurveillance studies and risk assessments [40]. Although no spillover events or outbreaks have yet been reported, we provide evidence for the potential of future spillover given the spatial overlap between bats and people in our study area. Even though there was no evidence for direct temporal overlap, the findings of Amman et al. [37] that viable Marburg virus can be detected on discarded fruit for up to 6 h illustrate the potential for indirect contact and exposure for humans. Specifically, we identify July as a high-risk period for spillover to humans as the potential for viral spillover is increased and bat activity in residential areas was highest during this period. Future human behavioural studies are planned to assess the behavioural patterns and activities of the villagers that may increase the risk of exposure and spillover. This could determine whether the degree of overlap between *R. aegyptiacus* from Matlapitsi cave and people is changing over time and will be paired with serological studies to determine whether spillover of bat-borne viruses has in fact occurred. Supplementary studies on the fruiting tree phenology in the area could identify whether bat movements into residential areas during the dry season is a predictable occurrence or if there are any phenological changes potentially caused by climate change. Another aspect for future consideration is the potential overlap between bats and livestock populations in the area given the historical evidence for livestock species to serve as intermediate hosts during spillover events [55].

## Methods

### Study area

**Fig. 4:**
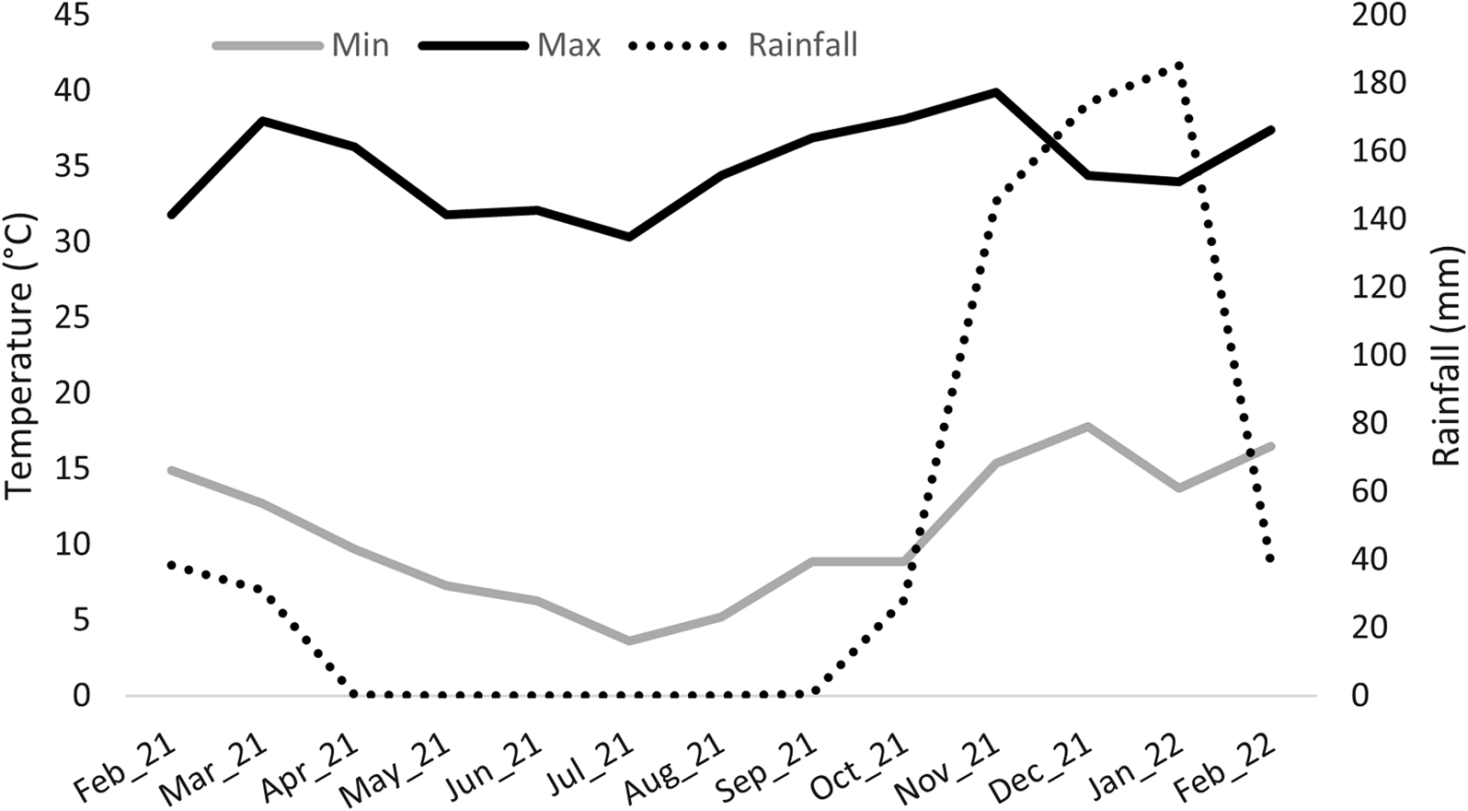
Weather station data. Weather station data for the Fertilis valley during the study period indicating the warm, wet and relatively cooler, dry seasons

**Fig. 5:**
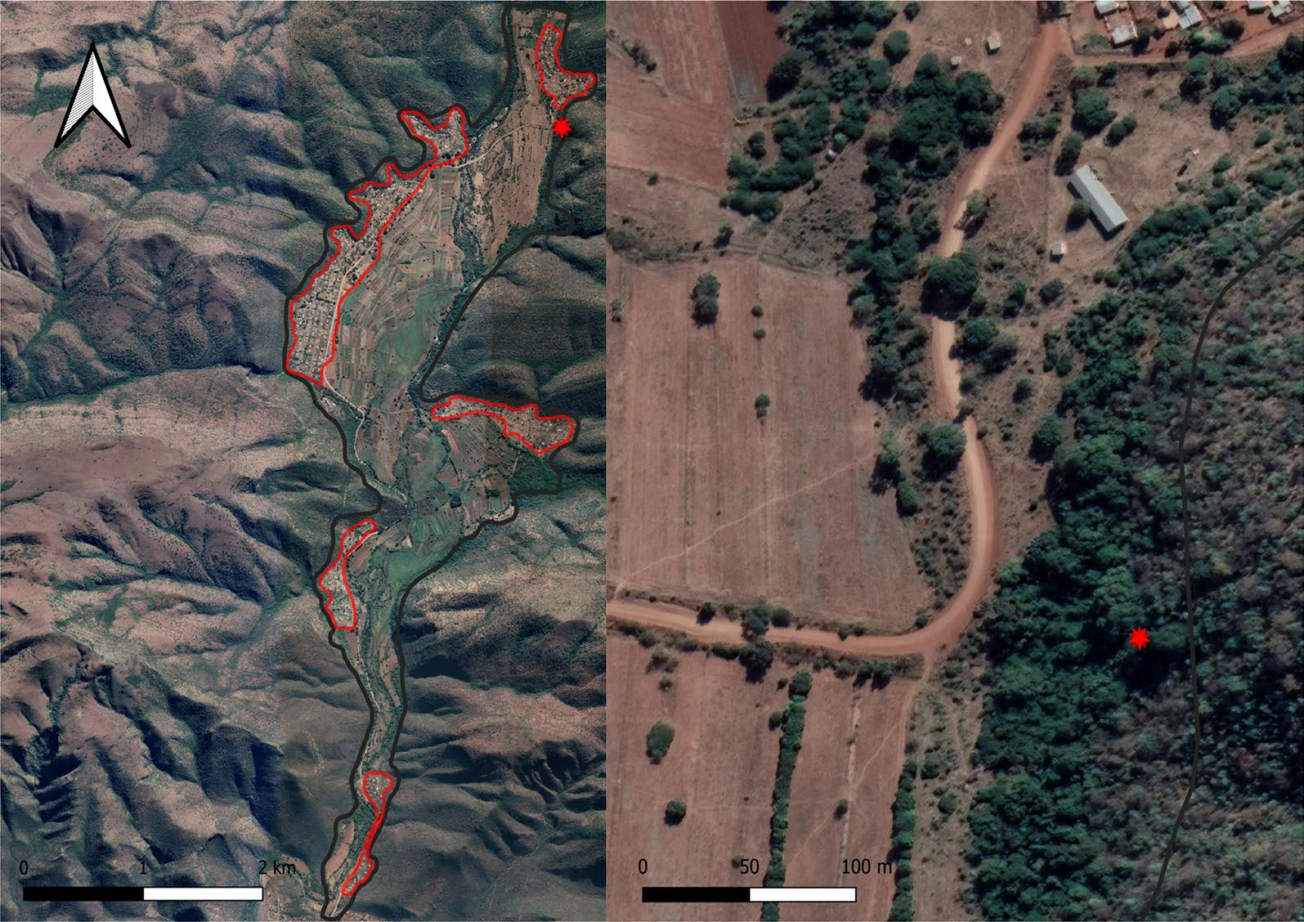
Study area and cave site. Satellite view of the Fertilis valley with the five major residential areas highlighted by the red encircled areas (left). Matlapitsi cave, as denoted by the red star, is situated in dense natural vegetation but is within 500 m of the nearest residential areas (right)

Fieldwork was performed around Fertilis, a rural settlement (24° 07′ 30″ S 30° 06′ 17″ E), north of the town of Ga Mafefe in Limpopo Province, South Africa. The area is located in a valley with an elevation ranging from 740 m a.s.l at the bottom of the valley to 990 m a.s.l on the mountain slopes. The climate can be described as typical humid subtropical with a warm, wet season from October until March and a relatively cooler, dry season from April until September (Fig. 4) (Additional file 7). The landscape contains large expanses of agricultural lands, which make up approximately 35% of the study area with patches of natural vegetation occurring along the Mohlapitsi river and on the mountain slopes, accounting for approximately 45% of the study area. Rural residential areas constitute the remaining 20% of landcover, with inhabitants residing in the five major residential areas (Fig. 5). Free-roaming livestock, including donkeys (*Equus asinus*), cattle (*Bos taurus*), goats (*Capra hircus*) and chickens (*Gallus domesticus*), are also prominent throughout the valley. A large proportion of households have fruiting trees on their properties including mangos (*Mangifera indica*)*,* papayas (*Carica papaya*)*,* litchis (*Litchi chinensis*)*,* avocados (*Persea americana*)*,* lemons (*Citrus limon*)*,* oranges (*C. sinensis*) and bananas (*Musa* spp.) which are used for subsistence. Despite the residents relying on these domestically grown fruiting trees, no protective measures were used to prevent foraging bats from eating the fruits. Native fruiting trees in the area include a variety of *Ficus* spp., including *Ficus sycomorus, F. sansibarica, F. ingens, F. abutilifolia* and *F. sur* as well as other trees such as Cape Ash (*Ekebergia capensis*) and Marula (*Sclerocarya birrea*)*.* Matlapitsi cave (24° 06′ 52″ S 30° 07′ 16″ E) is located in the northern section of the valley and serves as a maternity roost for an Egyptian rousette colony that is the focus of this study [33]. This cave has previously been used for religious and cultural practices and, although these have since been ceased, there is still evidence of people entering and exiting the cave (*personal observation*). The bat community residing within Matlapitsi cave have been the focal point for long-term virological studies, that started in 2012 [33], focusing on zoonotic disease in bats with ERBs from this colony being identified as hosts for Marburg virus and paramyxoviruses with zoonotic potential [30, 33, 35, 39].

### Small-scale movements

#### Capture and tagging

Bats were captured as they emerged from the cave using a 4.2 m^2^, 2-bank harp trap and a 1.2 m^2^ 3-bank harp trap (Faunatech Austbat, Bairnsdale, Victoria Australia). The traps were set up outside the cave entrance 60 min before sunset with the remainder of the cave entrance closed off by a tarpaulin to prevent bats from flying around the traps. All personnel operating the traps and handling the bats donned suitable Personal Protective Equipment (PPE) including disposable Tyvek® coveralls (Dupont™), a double pair of nitrile gloves (Lasec®) (the bottom layer of which is duct taped to the Tyvek coverall to create a sealed suit), handling gloves which are worn over the nitrile gloves, respiratory protection with Powered Air-Purifying Respirators (PAPR, Versaflow, Maxair, CAPR system) and gumboots (Bata Industrials, KwaZulu-Natal, South Africa). All individual bats were weighed, forearm length measured, and sex, age and reproductive status were determined. Age was determined by assessing the fusion of the epiphyseal gap of the phalangeal bones in the wing and based on forearm length measurements [70]. Bats with a forearm length greater than 89 mm were considered adults, whereas bats with a forearm length less than 89 mm were labelled as subadults. For reproductive status, females were separated into three categories: pregnant, non-pregnant and lactating, while males were separated into scrotal (having testes visibly descended into the scrotum) or non-scrotal.

A total of 26 bats were tagged, 15 males and 11 females. VHF radio transmitters (PD-2C, Holohil, Ontario, Canada) weighing 4.1 g were attached to the dorsal side of the bats with a collar and were also secured with a cyanoacrylate adhesive (Alteco Chemical Pte Ltd). The tag was positioned so that the transmitter sat between the scapulae and the antenna extended over the back and did not interfere with wing movements. We specifically did not tag pregnant females to avoid over-burdening them and risking injury. Collars were individually measured and fitted to each bat to ensure they would not fall off prematurely, or risk constriction around the neck. The collars were encased in Tygon® tubing to prevent injury through chafing and were connected by a small metal ferrule that was designed as a weak point enabling the eventual discarding of the tags after a few months, thus ensuring the tags would not remain permanently attached similar to the methods proposed by O'Mara et al. [71]. In cases where tags had fallen off and were recovered again, they were redeployed on newly captured bats using the same methods as described above. As the tags were designed to eventually fall off, the number of active tags varied each month (Table 6). The tags had an additional position-sensitive feature that enabled us to determine whether the bats were flying or not as the frequency of signal pulses doubled when the tag was parallel to the ground, indicative of flight. Bats were also tagged with Passive Integrated Transponders (PIT tags) to aid in identification should they be recaptured in the future. The total handling time for the bats, including measurements, PIT-tagging and collaring was less than 10 min.

**Table 6:** The number of active tags per month and the bat IDs of the *R. aegyptiacus* that were tracked each month

Year	Month	Active tags	Bat ID
2021	February	6	UP21-02; UP21-03; UP21-04; UP21-05; UP21-06; UP21-07
	March	6	UP21-02; UP21-03; UP21-04; UP21-05; UP21-06; UP21-07
	April	12	UP21-02; UP21-03; UP21-04; UP21-05; UP21-06; UP21-07; UP21-08; UP21-09; UP21-10; UP21-11; UP21-12; UP21-13
	May	10	UP21-02; UP21-03; UP21-05; UP21-07; UP21-08; UP21-09; UP21-10; UP21-11; UP21-12; UP21-13
	June	8	UP21-02; UP21-05; UP21-07; UP21-08; UP21-09; UP21-10; UP21-11; UP21-12
	July	6	UP21-02; UP21-10; UP21-11; UP21-12; UP21-14; UP21-15
	September	14	UP21-14; UP21-15; UP21-200; UP21-201; UP21-202; UP21-203; UP21-204; UP21-205; UP21-206; UP21-207; UP21-208; UP21-209; UP21-210; UP21-211; UP21-212
	October	12	UP21-200; UP21-201; UP21-202; UP21-203; UP21-204; UP21-205; UP21-206; UP21-207; UP21-208; UP21-209; UP21-210; UP21-211; UP21-212
	November	12	UP21-200; UP21-201; UP21-202; UP21-203; UP21-204; UP21-205; UP21-206; UP21-207; UP21-208; UP21-209; UP21-210; UP21-211; UP21-212
	December	12	UP21-200; UP21-201; UP21-202; UP21-203; UP21-204; UP21-205; UP21-206; UP21-207; UP21-208; UP21-209; UP21-210; UP21-211; UP21-212
2022	January	8	UP21-200; UP21-201; UP21-202; UP21-204; UP21-205; UP21-208; UP21-209; UP21-210
	February	5	UP21-200; UP21-201; UP21-202; UP21-204; UP21-209

Summary of the number of active tags per month

#### Tracking

Tags were calibrated with the receiver before deployment to allow for distance estimates by determining relative signal strengths at increasing distances away from the tags. The detection distance for tags, while still maintaining a reliable signal, was approximately 300 m. Once released, bats were tracked using a four-element Yagi antenna (VHF BNC 4-element Yagi, Africa Wildlife Tracking, Rietondale, Pretoria, South Africa) paired with a receiver (TR-4 Telonics, Africa Wildlife Tracking, Rietondale, Pretoria, South Africa). Tracking was performed by driving the available roads and walking transects in areas inaccessible to vehicles. Bats were tracked for between seven and nine nights each month, except for December 2021 where only five nights of tracking were performed due to Covid-19 cases affecting the research team. Furthermore, August 2021 was not sampled due to logistic constraints with tags not being available to deploy. Tracking started at sunset each night (weather dependent) and continued until an hour before sunrise. Once a bat was detected with the receiver, the distance to the bat was estimated based on signal strength, the bearing taken, and the GPS coordinates of the location recorded. Triangulation to determine the location of the bat was not possible as there was only a single tracking team. The bearing and distance estimates were used to estimate the approximate location of the bat with the following coordinate conversion calculations, where la1 = latitude of first point; la2 = latitude of second point; lo1 = longitude of first point; lo2 = longitude of second point; Ad = distance/Earth radius; ϴ = bearing in radians with bearing described as the clockwise angle from true north:

$$\begin{aligned} & \left( {{\text{la2}}} \right) \, = {\text{ arcsin}}({\text{sin}}\left( {{\text{la1}}} \right)*{\text{cos}}\left( {{\text{Ad}}} \right) \, + {\text{ cos}}\left( {{\text{la1}}} \right)*{\text{sin}}\left( {{\text{Ad}}} \right)*{\text{cos}}\left(^\circ \right) \\ & \left( {{\text{lo2}}} \right) \, = {\text{ lo1 }} + {\text{ arctan2}}({\text{sin}}\left(^\circ \right)*{\text{sin}}\left( {{\text{Ad}}} \right)*{\text{cos}}\left( {{\text{la1}}} \right),{\text{ cos}}\left( {{\text{Ad}}} \right) \, {-}{\text{ sin}}\left( {{\text{la1}}} \right)*{\text{sin}}\left( {{\text{la2}}} \right) \\ \end{aligned}$$

The outputs lo2 and la2 represent the approximate location of the bat that was detected. Additionally, the habitat type in which the bats were detected, and the activity of the bats were recorded. Bats were labelled as either ‘flying’ or ‘non-flying’ depending on signal impulse frequency. The decision to separate ‘flying’ from ‘non-flying’ activities is because there is a higher likelihood of activities linked to viral shedding occurring while bats are stationary. For example, while bats may urinate in flight, bats may urinate, defecate or discard fruit at foraging sites resulting in a higher concentration of potentially viral material than during flight. ERBs, however, do not feed while in flight [15, 38] and therefore, we can assume that non-flying activities equated to foraging or feeding while flying activities were attributed to commuting. Habitat was labelled as natural, agricultural or residential areas according to classifications from the SANLC dataset. In addition to recording the locations of the tagged bats, we searched for fruit bats and other wildlife with a spotlight by looking for eye shine while driving and these locations were also recorded.

### Fruit availability estimates

We walked line transects [72] to identify fruiting trees within the valley. A line transect is a simple method to survey biological populations whereby an observer walks an imaginary line counting individuals or objects within a certain distance from the line [73]. The locations for the transects were randomly selected within the valley, but we chose areas that represented all habitat types within the valley. We walked four transects at each location (terrain dependent) along the four cardinal directions (North, East, South and West). Each transect was 500 m long and all trees within 50 m on either side of the transect line were counted. Tree identification was performed with the aid of an identification guide [74]. For fruit availability estimates, we selected one fruiting tree of each species per transect, representing a variety of native and cultivated fruit trees, to serve as proxies for assessing seasonal variations in fruiting patterns for different species, in different areas throughout the study period (Additional file 4). Estimations were calculated as a percentage of total fruit cover, similar to the methods employed by Chapman et al. [75].

### Risk of contact and habitat selection

To assess the risk of contact between bats and people, all tracked locations for the bats were plotted onto a satellite map of the study area in QGIS (Quantum Geographic Information System) 3.12 [76] using the SANLC dataset to delineate the different habitat types. The dataset was downloaded from the South African Department of Environmental Affairs (DEA) Environmental Geographic Information Systems (EGIS) website (https://egis.environment.gov.za/data_egis/data_download/current). The proportion of locations obtained for bats within residential areas was calculated each month similar to the method used by Randhawa et al. [23] where they included locations within 100 m of urban areas in their risk assessment. We assessed the proportion of all locations within residential areas but also specifically assessed non-flying locations as these represent locations where bats are likely to spend longer durations which could increase the risk of direct and indirect contact. The proportional cover of different habitat types within the study area was estimated using the landcover map which enabled us to quantify the proportional usage of each landcover type by the bats each month.

### Statistical analyses

#### Movements

We performed Generalised Linear Mixed Models (GLMMs) with Poisson distribution and log link function to analyse the number of locations obtained each month for different habitat types and activities and whether there were significant differences across the study period. Model selection was performed using the ‘lme4’ and ‘AICcmodavg’ packages in R [77, 78] through backward, stepwise logistic regression where candidate models were generated by sequentially removing variables from the full model until the best-performing model was obtained. We included habitat type (residential/agricultural/natural), the activity of the bat (flying/non-flying) and season (dry/wet) as fixed effects with month as a random effect. Model performance was assessed with corrected Akaike’s Information Criterion (AICc) values, with the lowest AICc value corresponding to the best-performing model. Models that were within ΔAICc < 2 were considered as competing [79]. Results from the models were interpreted using the ‘lmertest’ [80] and ‘emmeans’ [81] packages with marginal and conditional R^2^ values reported from the MuMIn package [82].

We were unable to include variables linked to sampling effort in the main GLMMs as the number of nights, hours tracked and the number of active tags were specific to each month and as such there was strong multicollinearity between these variables and the study month as well as singularities in the dataset which would confound the analysis. Therefore, to assess whether sampling effort influenced the number of locations obtained each month, we ran additional separate linear models. These models included the number of nights, hours tracked and active tags each month as independent variables.

#### Habitat usage and resource selection

**Fig. 6:**
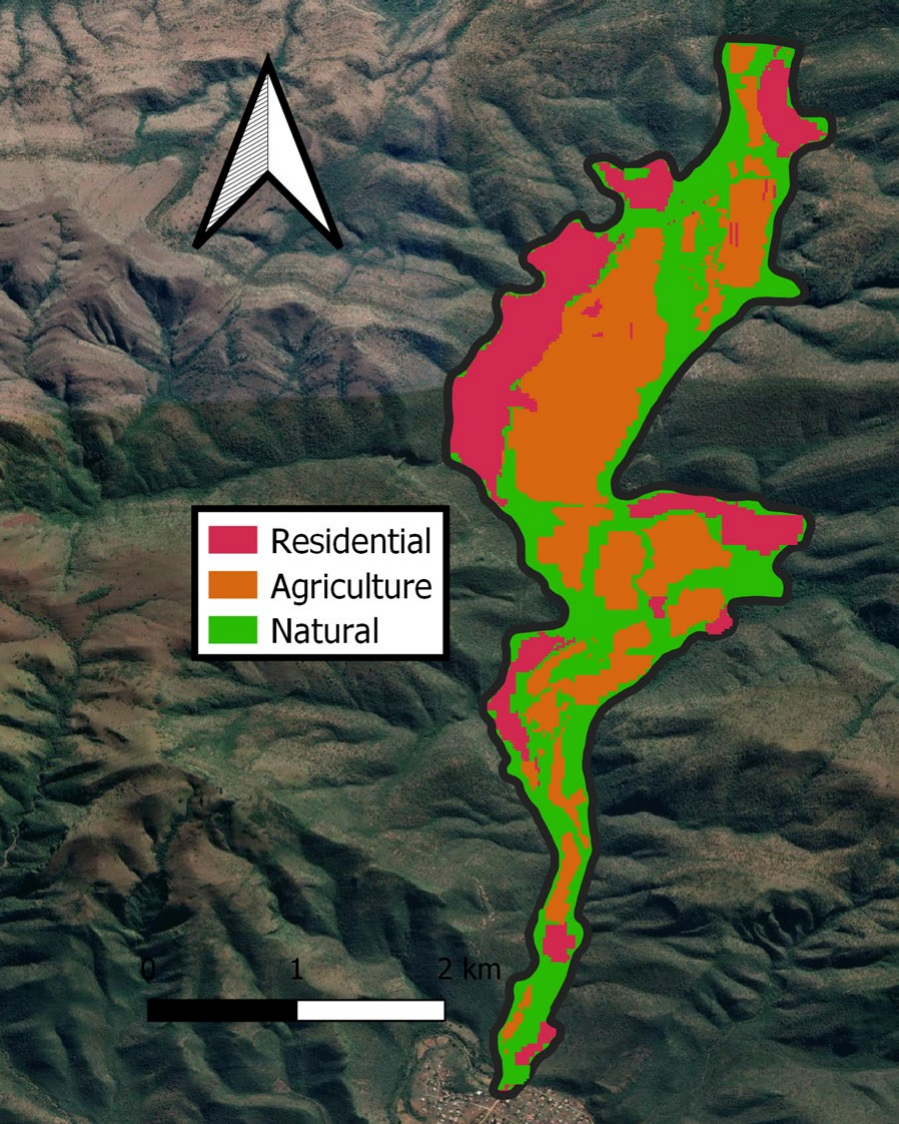
Landcover classification map. Satellite image of the Fertilis valley detailing the different habitat types obtained from the SANLC dataset

Habitat usage and resource selection were assessed using the landcover maps with the tracked locations plotted onto the maps in QGIS. Habitat types with similar classifications were concatenated into single classes. For example, formal residential, informal residential and scattered village areas were all grouped as residential areas. After simplifying the habitat classifications, three distinct habitat classes remained: residential, natural and agricultural areas (Fig. 6). The area of each of these habitats was measured as well as the area of the whole study area using polygons in QGIS to obtain an estimate of proportional availability for each habitat type in the study area. After this, the number of locations within each habitat was counted and compared against the proportion of habitat availability in the study. We performed a resource selection ratio analysis using the ‘adehabitatHS’ package v 3.15 in R [83] using the widesI analysis as the data were pooled across individuals with the same proportion of habitat available in all cases [84]. Selection ratios were performed for the different activities (flying/non-flying) as well as between the sexes to determine if there were any preferences apparent between the categories.

#### Utilization distributions

As a further metric of habitat usage each month, we calculated utilization distributions using kernel density estimates [85] for bat movements each month. Although we had sufficient points to calculate home range estimates for several individual bats [86], the layout of the study area prevented us from tracking the bats if they left the valley and therefore, any home range estimations would be heavily biased towards our study area. Therefore, the utilization distributions were calculated to identify specific areas of usage rather than to estimate home range sizes. Location data were first projected into the Universal Transverse Mercator coordinate system for ease of analysis and import into QGIS. Kernel density estimates were calculated using the ‘adehabitatHR’ package v 4.19 in R [83] using the least squares cross-validation (LSCV) method for selecting bandwidth [87]. Kernel estimates generate contours around a pre-specified proportion of points and for our analysis, we calculated density estimates for 50, 60, 70, 80, 90 and 95% locations. This was done to investigate patterns of habitat usage with increasing specificity and to identify any anomalous trends. The kernels obtained from the analysis were overlayed with the satellite map of our study site and the proportional usage of different habitat types was calculated as the area for each habitat encompassed by the kernels. Areas were calculated using polygons in QGIS. We assessed usage across all three habitat types and compared the proportional usage between wet and dry seasons for non-flying and all locations to determine whether usage differed significantly between seasons, for specific activities or different habitat types. We assessed differences in proportional usage between seasons or for non-flying activities using paired t-tests. All data included in the tests were normally distributed according to Shapiro–Wilk tests. All analyses were performed in R v4.0.2 [88] using the RStudio v1.3.1073 [89] interface and statistical significance was assessed at an alpha threshold of 0.05 unless otherwise stated.

## Supplementary Information

**Additional file 1**. Tracking data. Location data for the 26 tracked bats and bat sightings in the Fertilis valley.

**Additional file 2**. Model selection. Summary of candidate models used for the analysis.

**Additional file 3**. Comparison of residential area usage. Percentage of foraging and all locations in residential areas.

**Additional file 4**. Vegetation data. Fruit availability estimates for selected fruiting trees in the Fertilis valley.

**Additional file 5**. Comparison of foraging areas. Paired *t* tests assessing proportional area size utilised for foraging activities within each habitat type during July 2021 and January 2022.

**Additional file 6**. Utilization distribution sizes. Comparison of the proportional area sizes within the different habitat types for foraging activities in July 2021 and January 2022.

**Additional file 7**. Weather data. Weather station data from the Fertilis valley for the duration of the study period.

## Data Availability

The datasets supporting the conclusions of this article are included within the article and its additional files.

## Abbreviations

AEC: Animal Ethics Committee
AICc: Corrected Akaike’s Information Criterion
a.s.l: Above sea-level
BEEZ: Biosurveillance and Ecology of Emerging Zoonoses
BNC: Bayonet Neill-Concelman
CVZ: Centre for Viral Zoonoses
DEA: Department of Environmental Affairs
DSI: Department of Science and Innovation
EGIS: Environment Geographic Information Systems
GLMM: Generalised Linear Mixed Model
GPS: Global Positioning System
ha: Hectares
KZN: KwaZulu-Natal
LSCV: Least squares cross-validation
NRF: National Research Foundation
PAPR: Powered air-purifying respirators
PIT: Passive Integrated Transponder
PPE: Personal Protective Equipment
QGIS: Quantum Geographic Information System
RNA: Ribonucleic acid
SANLC: South African National Landcover
VHF: Very high frequency
