# Supplementary material for: Variations in small-scale movements of, Rousettus aegyptiacus, a Marburg virus reservoir across a seasonal gradient
Source: Front Zool. 2023 Jul 18;20:23. doi: 10.1186/s12983-023-00502-2 (PMC10353151; doi:10.1186/s12983-023-00502-2)
Supplement: Supplementary file 2 — Additional file 2. Model selection. Summary of candidate models used for the analysis. [file 12983_2023_502_MOESM2_ESM.docx]

Table 2: Model selection

| Model | Candidate models | K | AICc | ΔAICc | AICc.Wt |
| --- | --- | --- | --- | --- | --- |
| 1 | Locations ~ Habitat*Activity + (1\|Month) | 7 | 426.27 | 0.00 | 0.656 |
| 2 | Locations ~ Habitat*Activity + Season + (1\|Month) | 8 | 427.92 | 1.65 | 0.287 |
| 3 | Locations ~ Habitat + (1\|Month) | 4 | 432.46 | 6.19 | 0.030 |
| 4 | Locations ~ Habitat + Activity + (1\|Month) | 5 | 433.87 | 7.60 | 0.015 |
| Full | Locations ~ Habitat + Activity + Season + (1\|Month) | 6 | 435.37 | 9.10 | 0.007 |
| 6 | Locations ~ Habitat + Activity*Season + (1\|Month) | 7 | 435.98 | 9.71 | 0.005 |
| 7 | Locations ~ Habitat*Season + Activity + (1\|Month) | 8 | 438.79 | 12.52 | 0.000 |
| Null | Locations ~ 1 + (1\|Month) | 2 | 565.85 | 139.58 | 0.000 |
| 9 | Locations ~ Activity + (1\|Month) | 3 | 567.12 | 140.85 | 0.000 |

Summary of candidate models with full and null models for comparison with AICc scores, model weight (AICcWt) and parameters (K).
