# Supplementary figures and images for: Variations in small-scale movements of, Rousettus aegyptiacus, a Marburg virus reservoir across a seasonal gradient
Source: Front Zool. 2023 Jul 18;20:23. doi: 10.1186/s12983-023-00502-2 (PMC10353151; doi:10.1186/s12983-023-00502-2)

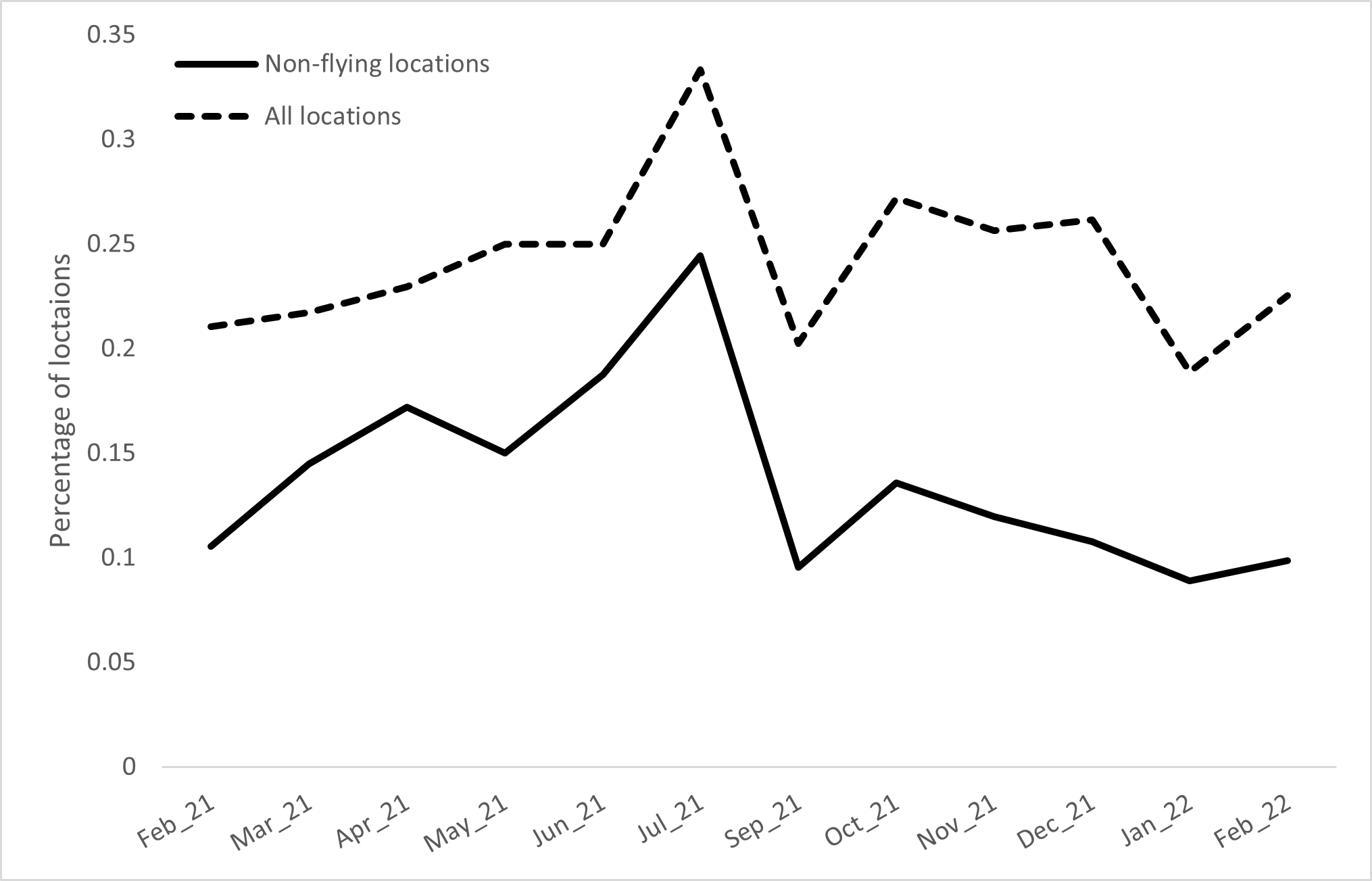

Supplement: Supplementary file 3 — Additional file 3. Comparison of residential area usage. Percentage of foraging and all locations in residential areas. [file 12983_2023_502_MOESM3_ESM.png]
