# Supplementary material for: Variations in small-scale movements of, Rousettus aegyptiacus, a Marburg virus reservoir across a seasonal gradient
Source: Front Zool. 2023 Jul 18;20:23. doi: 10.1186/s12983-023-00502-2 (PMC10353151; doi:10.1186/s12983-023-00502-2)
Supplement: Supplementary file 5 — Additional file 5. Comparison of foraging areas. Paired t tests assessing proportional area size utilised for foraging activities within each habitat type during July 2021 and January 2022. [file 12983_2023_502_MOESM5_ESM.docx]

Table 7: Pairwise comparison of area usage for foraging activities

| Comparison | Estimate | t-value | p-value |
| --- | --- | --- | --- |
| **Jan.Agricultural < Jan.Natural** | **-1.74** | **-3.35** | **0.020** |
| **Jan.Agricultural > Jan.Residential** | **7.76** | **5.90** | **0.002** |
| **Jan.Agricultural > Jul.Agricultural** | **5.62** | **3.94** | **0.011** |
| **Jan.Agricultural > Jul.Natural** | **8.29** | **4.74** | **0.005** |
| **Jan.Agricultural < Jul.Residential** | **-17.48** | **-6.41** | **0.001** |
| **Jan.Natural > Jan.Residential** | **9.49** | **5.35** | **0.003** |
| **Jan.Natural > Jul.Agricultural** | **7.35** | **3.89** | **0.011** |
| **Jan.Natural > Jul.Natural** | **10.02** | **4.96** | **0.004** |
| **Jan.Natural < Jul.Residential** | **-15.75** | **-5.90** | **0.002** |
| **Jan.Residential < Jul.Agricultural** | **-2.14** | **-3.18** | **0.024** |
| Jan.Residential – Jul.Natural | 0.53 | 0.42 | 0.693 |
| **Jan.Residential < Jul.Residential** | **-25.24** | **-7.35** | **<0.001** |
| Jul.Agricultural – Jul.Natural | 2.67 | 1.42 | 0.214 |
| **Jul.Agricultural < Jul.Residential** | **-23.10** | **-7.56** | **<0.001** |
| **Jul.Natural < Jul.Residential** | **-25.77** | **-5.93** | **0.002** |

Paired t-tests assessing proportional area size utilised for foraging activities within each habitat type during July 2021 and January 2022 with mean difference estimates, t-values and p-values.
