# Supplementary material for: Variations in small-scale movements of, Rousettus aegyptiacus, a Marburg virus reservoir across a seasonal gradient
Source: Front Zool. 2023 Jul 18;20:23. doi: 10.1186/s12983-023-00502-2 (PMC10353151; doi:10.1186/s12983-023-00502-2)
Supplement: Supplementary file 6 — Additional file 6. Utilization distribution sizes. Comparison of the proportional area sizes within the different habitat types for foraging activities in July 2021 and January 2022. [file 12983_2023_502_MOESM6_ESM.docx]

Table 8: Utilization distribution area sizes

|  | July 2021 | | | | January 2022 | | |
| --- | --- | --- | --- | --- | --- | --- | --- |
|  | Agriculture | | Natural | Residential | Agricultural | Natural | Residential |
| 50% | 3.71 | 6.76 | | 16.24 | 7.59 | 9.08 | 3.61 |
| 60% | 8.48 | 10.28 | | 28.69 | 16.58 | 17.90 | 8.09 |
| 70% | 17.07 | 16.25 | | 45.58 | 27.93 | 31.75 | 15.15 |
| 80% | 35.18 | 29.28 | | 68.20 | 41.56 | 44.15 | 31.81 |
| 90% | 55.67 | 48.17 | | 81.78 | 58.65 | 59.50 | 52.83 |
| 95% | 67.62 | 60.98 | | 85.86 | 69.13 | 69.48 | 63.41 |
| Average | 31.28 | 28.62 | | 54.39 | 36.91 | 38.64 | 29.15 |

Comparison of the proportional area sizes in hectares (ha) within the different habitat types for foraging activities in July 2021 and January 2022. Area sizes were calculated for kernels with a different proportion of locations ranging from 50 – 95%.
